# Preventive interventions to reduce the burden of rheumatic heart disease in populations at risk: a systematic review protocol

**DOI:** 10.1186/s13643-021-01748-9

**Published:** 2021-07-08

**Authors:** Panduleni Penipawa Shimanda, Tonderai Washington Shumba, Mattias Brunström, Stefan Söderberg, Lars Lindholm, Scholastika Ndatinda Iipinge, Fredrik Norström

**Affiliations:** 1 Department of Epidemiology and Global Health, Umeå University, SE 901 87 Umeå, Sweden; 2 Clara Barton School of Nursing, Welwitchia Health Training Centre, P. O. Box 98604, Pelican Square, Windhoek, Namibia; 3 Department of Occupational Therapy and Physiotherapy, School of Allied Health, University of Namibia, Windhoek, Namibia; 4 Department of Public Health and Clinical Medicine, Umeå University, SE 901 87 Umeå, Sweden

**Keywords:** Acute Rheumatic Fever, Rheumatic Heart Disease, Intervention, Prevention, Systematic Review

## Abstract

**Background:**

Rheumatic heart disease is preventable, yet associated with significant health burden, mostly in low-resourced settings. It is prevalent among children and young adults living in impoverished areas. Primordial, primary, and secondary preventive measures have been recommended through health interventions and comprehensive programmes, although most implemented interventions are the high-resourced settings. The proposed review aims to synthesise the evidence of prevention effectiveness of implemented health interventions for the prevention of rheumatic heart disease.

**Methods and design:**

This article describes a protocol for a systematic review. A predefined search strategy will be used to search for relevant literature published from the year 2000 to present. Electronic databases Medline, Web of Science, Scopus, and Cochrane Central Register of Controlled Trials will be searched for the studies, as well as reference lists of relevant studies included. Risk of bias and quality appraisal will be done for the included studies using ROBINS-I tool and Cochrane tool for assessing risk of bias in randomised control trials. Findings will be analysed in subgroups based on the level of intervention and prevention strategy implemented. We will present the findings in descriptive formats with tables and flow diagrams.

**Discussion:**

This review will provide evidence on the prevention effectiveness of interventions or strategies implemented for the prevention of RHD. The findings of this will be significant for policy, practice, and research in countries planning to implement interventions.

**Registration:**

PROSPERO ID: CRD42020170503.

**Supplementary Information:**

The online version contains supplementary material available at 10.1186/s13643-021-01748-9.

## Background

Rheumatic heart disease (RHD) is a preventable non-communicable disease which is mostly prevalent among children and young adults and potentially fatal among these groups. It is a sequela of acute rheumatic fever (ARF), which is most common in poor settings associated with overcrowding, poor sanitation, and other social determinants of health. ARF results from an autoimmune response to pharyngitis caused by group A streptococcus infection [1, 2].

The Global Burden of Disease have estimated that by 2019 about 40.5 million people live with RHD globally, causing 306 000 deaths per annum and 8.7 million disability-adjusted life-years. The prevalence is noted to be rising steadily. Although, the overall global burden remains unequally skewed towards low-resource settings, mainly in Oceania, South Asia, and sub-Saharan Africa [3, 4]. In these low-resource settings, RHD is prevalent among young people, and mostly women of childbearing age [5].

RHD can be prevented, mainly by the three approaches: primordial, primary, and secondary prevention. Primordial prevention targets to reduce the risk of group A streptococcal pharyngitis by addressing underlying social determinants of health and improving access to health care. Primary prevention can be achieved through effective treatment of group A streptococcal pharyngitis with penicillin antibiotics to avoid acute rheumatic fever. Secondary prevention is a continuous administration of penicillin to people with a history of rheumatic fever and/or rheumatic heart disease to prevent streptococcal pharyngitis and recurrence of rheumatic fever [2, 6–8].

Proper prevention of RHD, toward eradication, will significantly reduce the burden of mortality by cardiovascular diseases worldwide. Resulting, RHD is given a high priority on the international agenda, with actionable strategies developed and recommendations for endemic states, including key statements from Africa [9–12].

Strong recommendations are placed toward comprehensive programmes harnessing preventative and control synergies to combat RHD, while ensuring such programmes are integrated into existing national health systems [12]. Despite the recommendations, there is paucity of evidence on current practices, how they vary across settings, and the emerging practices for preventing and reducing the burden of RHD. Reported inspirational and successful programmes that demonstrated effectiveness in reducing the burden of ARF and RHD were implemented in the 1980s and 1990s [9, 13–15]. Considering the epidemiological transition and socioeconomic development over the recent decades [16], it is necessary to synthesise and report contemporary information on interventions aiming to prevent and reduce the global burden of RHD.

Synthesising the current evidence will guide further development of interventions in endemic settings, also giving a view on the progress of countries towards global RHD goals for control and eradication.

A recent review and meta-analysis on the integration of prevention and control programmes for RHD in country health systems mainly included programmes implemented 20–30 years ago [17]. While the review only focused on comprehensive programmes, a gap remains on the effects of generic interventions implemented not as comprehensive programmes. These could, for instance, focus on a single preventive strategy in resource-limited settings where comprehensive programmes are not feasible.

In this review, we define intervention as any preventive measure implemented that seeks to reduce the risk and/or burden of RHD in a population. Our review aims to provide current information on the effect of interventions of care implemented, how these interventions vary globally, and identify emerging practices implemented in the prevention and reduction of the burden of RHD in endemic countries in the twenty-first century.

The objective of the study is to synthesise the available literature to identify interventions implemented to reduce the burden of rheumatic heart disease and assess their prevention effectiveness. Interventions on primordial, primary, and secondary prevention strategies will be considered at all levels (patient, community, and health system). We will put an emphasis on identifying emerging practices in the preventive interventions for rheumatic heart disease implemented from the year 2000 to date.

## Method design

The review process will be guided by the Cochrane Collaborations Handbook for systematic reviews [18]. The protocol is developed in compliance with the Preferred Reporting Items for Systematic Review and Meta-Analysis Protocols (PRISMA-P) guidelines [19], and the final review will be reported following the PRISMA format [20]. The protocol is registered within the International Prospective Register of Systematic Reviews (PROSPERO) - PROSPERO ID: CRD42020170503, available at: https://www.crd.york.ac.uk/PROSPERO/display_record.php?RecordID=170503. Ethical approval is not necessary, given this is a systematic review, synthesising previously published data on an aggregate level. A PRISMA-P checklist is compiled for this protocol (Annexure 1).

### Criteria for considering studies for this review

#### Type of studies

We will consider both experimental and observational studies reporting the effects of interventions. These will include randomised controlled trials, clinical controlled trials, cluster randomised trials, quasi-experimental studies, and cross-sectional studies. With an anticipated paucity of comparative studies in the area of study, relevant descriptive studies will be considered for inclusion if reporting on the implementation, feasibility, and coverage of the intervention.

#### Interventions

The intervention of interest includes an objective on the prevention of streptococcal pharyngitis, ARF, and RHD. This will broadly include studies that describe activities performed towards (1) reduction of poverty, inequalities, or improving access to health care (primordial prevention), (2) knowledge and awareness creation, early detection, and treatment of pharyngitis (primary prevention), and (3) strengthening of antibiotic prophylaxis among people with ARF/RF and RHD (secondary prevention).

The interventions will be stratified into themes: patient, community, and health system levels. We would expect some of the interventions to be targeting two or more system levels and as well different prevention levels.

#### Type of participants

In the patient-level interventions, the unit of analysis will be individuals at risk of, or affected by, streptococcal pharyngitis, rheumatic fever, and rheumatic heart disease. Community-level interventions will have communities, schools, regions, districts, etc. as the unit of analysis. Health system-level interventions will use healthcare workers proving care relating to rheumatic heart disease, service-delivery improving strategies as the unit of analysis. There will be no limitation regarding participants’ socio-demographic characteristics.

#### Outcome of interest

Study results must include quantitative data for outcomes measured. The review will aim to describe intervention outcomes as compared to a control group with a different intervention, pre-intervention, or location/population with no intervention. Therefore, the primary outcome of interest will be considered: less streptococcal pharyngitis (primordial), less ARF cases (primary), and less RHD (secondary). We will contact authors to obtain the full article if an interest is found and we do not have full article access. Further, authors will be contacted for data if a relevant study reporting on an intervention is found with missing data or ambiguous reporting of outcome.

#### Timing

We propose to review and report on studies published from the year 2000 to present for two reasons [1]; after a period of neglection, key global and regional RHD-specific resolutions and policy activities were again brought into action by 2005, as summarised in Abouzeid et al. [21] [2]. We hypothesise that many countries have better response and implementation of RHD resolutions in the twenty-first century, considering the socioeconomic development as compared to the twentieth century. The epidemiological transition is another factor we consider having influenced and pushed RHD into countries’ non-communicable disease plans in the tweny-first century.

#### Setting

Studies conducted globally will be included.

#### Language

No language restriction will be applied.

### Exclusion criteria

- Studies will be excluded if they do not display a clear effort to practice preventive services to people at risk or affected by streptococcal pharyngitis, ARF/RF, and RHD.

### Search strategy

A comprehensive electronic literature search among the main electronic databases (Medline, Web of Science, Elsevier’s Scopus, EMBASE, Cochrane Central Register of Controlled Trials, CINAHL) will be conducted to identify relevant literature. Search strategies will be tailored to meet the requirements of each electronic database (Annexure 2). The search strategy will be constructed using Medical Subject heading and text words in relation to keywords “pharyngitis”, “rheumatic fever”, “rheumatic heart disease”, “prevention”, and “intervention”.

Reference lists of included studies will be hand-searched to review for relevant studies missed in the electronic searches.

### Selection of studies

Search results will be imported and managed using Endnote X9 software (Clarivate Analytics, Philadelphia, PA, USA). Two reviewers (PS and TS) will independently screen titles and abstracts of all articles identified to select potentially eligible studies as per the predefined inclusion and exclusion criteria. Full-text versions of all potentially eligible studies will be reviewed for eligibility. Results will be shared and variances discussed to reach consensus. A third reviewer will be consulted when consensus cannot be reached between the two reviewers.

In cases full text is not available, it will be obtained through the Umeå university library or the corresponding author will be contacted. Search processes, selection of studies, and rationale for exclusions will be summarised and presented in flow diagrams applying the PRISMA guidelines.

### Data extraction and management

Data will be extracted from the selected studies and recorded in pre-designed forms. Descriptive information will be recorded in a Microsoft Word 2019 form (Annexure 3), while numeric data will be recorded in a Microsoft Excel 2019 spreadsheet.

The data extraction form will capture for objective one (section C, D & E) [1]; basic study characteristics, including objectives, study population, sample size, years and location of study and study design [2]; details of intervention(s), health outcomes of the intervention, impacts of the intervention, and resources used. For objective two data will be captured on emerging practices (section F) in the prevention of RHD such as integration with other chronic noncommunicable disease programmes. This will highlight effective measures being implemented compared to disease-specific traditional interventions.

Full-text studies will be tentatively categorised into prevention groups during the review process: (1) related to primordial prevention, (2) primary prevention, and (3) secondary prevention. Studies will also be analysed according to the methodology background, and the level of intervention investigated in the study.

Two reviewers will independently extract the data using the extraction forms. All data extracted will be compared between the authors and double-checking against the original publication if any discrepancies. A third reviewer will be consulted if discrepancies still remain after discussions.

For studies found to have unclear or missing data, the corresponding author will be contacted to clarify the findings.

### Risk of bias and quality appraisal of included studies

Assessment for risk of bias will be performed for all studies meeting inclusion criteria in terms of their design methodology, biases, and confounders. Two authors will independently assess the methodological quality of each study in accordance with the methods recommended by the Cochrane Collaboration.

Studies selected for inclusion will be critically appraised in terms of their design methodology and biases. The Cochrane Collaboration’s tool will be used to assess the risk of bias of randomised control trial studies [22]. The criteria to assess the risk of bias in randomised controlled trials will cover the main domains: bias arising from randomisation process, bias due to deviations from intended interventions, bias due to missing outcome data, bias in measurement of the outcome, and bias in selection of the reported result. The risk of bias and potential confounders in observational studies will be assessed using the ROBINS-I tool [23] (Annexure 4). Pre-intervention, At-intervention, and Post-intervention bias domains will be considered while assessing for relevant categories of bias in each domain. Important potential confounders anticipated in included studies are socioeconomic factors, and implementation strategies. Information will be documented on which judgements are based. To minimise the risk of publication bias, we will search for and include relevant grey literature studies in clinical registries, regulatory agency websites, and conference abstracts. Experts will be contacted for possible unpublished studies based on relevant identified grey literature. Symmetry funnel plots will be used to assess meta-biases if a significant number of eligible studies are identified.

### Data synthesis and presentation

The review will synthesise study data using qualitative and quantitative approaches. Studies will be grouped based on the level of intervention: patient, community, and health system. Within these groups, subgroups will be created based on the prevention strategy and unit of analysis (population/community/participants). Findings will be presented in narrative format, including suitable tables and figures, with effectiveness measures such as relative and absolute differences. To report the effect measures, risk ratio/odds ratio/risk difference with corresponding 95% confidence intervals will be used for dichotomous data, while mean difference and standard deviations will be used for continuous data. Standardised mean differences will be used if outcomes are reported using different scales. For observational studies, adjusted data will be used in the analyses while taking into consideration factors adjusted for in the study. Evidence of included studies will be assessed and graded as very low, low, moderate, and high quality using the Grading Recommendations Assessment, Development and Evaluation (GRADE) approach [24]. If the included studies are sufficiently homogenous (relating to study population, methodology, intervention, and outcome) meta-analyses will be considered, using random-effects model to account for between-study variability. If a meta-analysis is conducted statistical heterogeneity will be assessed using the *X*^2^ test having a 10% significance level and quantified using the *I*^2^ statistic.

### Dissemination of findings

The findings of this review will be broadly disseminated via conference presentations and peer-reviewed publications.

## Discussion

### Expected significance of the study

The prevention and management of ARF and RHD in endemic areas has been well outlined in the Tools for Implementing Rheumatic Heart Disease Control Programmes (TIPs) Handbook [25]. The handbook broadly focused on comprehensive programmes, drawing from evidence from the historical studies of comprehensive programmes implemented mostly in the 1950s–1980s. Although comprehensive programmes are recommended, RHD is prevalent in low-resource settings where policy makers are likely required to make trade-offs between different interventions, only affording to implement specific preventive strategies and not comprehensive programmes. This review aims to bridge the gap in knowledge by documenting the effect of specific interventions or strategies for the prevention of RHD. The findings of this review will be significant for policy, practice, and research in countries planning to implement interventions. Evidence of effective strategies may help policy markers make trade-offs between strategies to implement when comprehensive programmes are not feasible. In addition, our results will present evidence of practices of RHD prevention implemented in the twenty-first century, also aiming to identify emerging practices.

### Potential limitations of review methods

We anticipate challenges in this review. The scope is broad, and we expect a big divergence in studies. We may also be limited by the methodology of the included studies. The possibilities to make causal inferences from studies of, for example historical controls of comparisons between regions, are limited. Consequently, meta-analyses are not likely to be motivated.

## Supplementary Information

**Additional file 1: Annexure 1.**

**Additional file 2: Annexure 2.**

**Additional file 3: Annexure 3.**

**Additional file 4: Annexure 4.**

## Data Availability

Not applicable.

## Abbreviations

ARF: Acute rheumatic fever
GRADE: Grading of Recommendations Assessment, Development, and Evaluation
PRISMA: Preferred Reporting Items for Systematic Reviews and Meta-Analyses
PRISMA-P: Preferred Reporting Items for Systematic Reviews and Meta-Analyses Protocols
RF: Rheumatic fever
RHD: Rheumatic heart disease
