# Supplementary material for: Preventive interventions to reduce the burden of rheumatic heart disease in populations at risk: a systematic review protocol
Source: Syst Rev. 2021 Jul 8;10:200. doi: 10.1186/s13643-021-01748-9 (PMC8268600; doi:10.1186/s13643-021-01748-9)
Supplement: Supplementary file 2 — Additional file 2: Annexure 2. [file 13643_2021_1748_MOESM2_ESM.docx]

**Annexure 2: Search strategy**

| **#** | **Database** | **Query** | **Results** | **Date of query** |
| --- | --- | --- | --- | --- |
| 1 | PubMed/Medline | ((((“Rheumatic fever”[mesh] OR “rheumatic fever”[tiab] OR "rheumatic heart disease"[mesh] OR “rheumatic heart disease”[tiab] OR “acute rheumatic fever”[tiab] OR “pharyngitis”[tiab] OR “sore throat”[tiab] OR “group A strep*”[tiab] OR RHD[tiab]))) AND prevent*[tiab]) AND ((interve*[tiab] OR program*[tiab])) Sort by: Best Match Filters: Publication date from 2000/01/01 | 326 | 07/05/2020 |
| 2 | Web of Science (Time filter 2000-2020, Search all databases) | TS=(Rheumatic fever OR rheumatic heart disease OR acute rheumatic fever OR pharyngitis OR sore throat OR group A strep* OR RHD) AND TS=(intervention OR program*) AND TS=(prevent*) | 1984 | 07/05/2020 |
| 3 | Scopus | ( TITLE-ABS-KEY ( "Rheumatic fever" OR "rheumatic heart disease" OR "acute rheumatic fever" OR "pharyngitis" OR "Sore throat" OR "group A strep*" OR "RHD" ) AND TITLE-ABS-KEY ( prevent* ) AND TITLE-ABS-KEY ( "interve*" OR program* ) ) AND PUBYEAR > 1999 | 726 | 07/05/2020 |
