# Supplementary material for: Preventive interventions to reduce the burden of rheumatic heart disease in populations at risk: a systematic review protocol
Source: Syst Rev. 2021 Jul 8;10:200. doi: 10.1186/s13643-021-01748-9 (PMC8268600; doi:10.1186/s13643-021-01748-9)
Supplement: Supplementary file 3 — Additional file 3: Annexure 3. [file 13643_2021_1748_MOESM3_ESM.docx]

**Annexure 3: Data extraction form**

| Study ID (first author surname and year of publication) |
| --- |
|  |

**Section A: General information**

| Date form completed  (dd/mm/yyyy) |  |
| --- | --- |
| Name of data extractor |  |
| Reference citation |  |
| Study author contact details |  |
| Publication type  (e.g. full report, abstract) |  |
| Potentially eligible studies found in the reference list |  |
| Notes: | |

**Section B: Eligibility criteria**

| **Study characteristics** | **Eligibility criteria** | **Criteria met?** |
| --- | --- | --- |
| Type of study | RCT, CCT, CBA, ITS, quasi-experimental, cross-sectional | Yes  No  Unclear |
| Intervention objectives | Prevention of streptococcal pharyngitis, ARF, RHD | Yes  No  Unclear |
| Types of outcome measures | Intervention outcomes | Yes  No  Unclear |
|  | Intervention characteristics | Yes  No  Unclear |
| Include  Exclude  Pending | | |
| Rationale for exclusion |  | |
| Notes: | | |

**DO NOT PROCEED IF STUDY EXCLUDED FROM REVIEW**

**Objective one**

**Section C: Characteristics of included studies**

|  | Description as stated in the paper/report |
| --- | --- |
| Objective(s) of study |  |
| Brief description of intervention(s) studied |  |
| Study design |  |
| Country, province/state/region |  |
| Population/Community |  |
| Notes: | |

**Section D: Outcome measures:**

**I. Intervention characteristics**

|  | Description as stated in the paper/report |
| --- | --- |
| Start year |  |
| Duration |  |
| Level of intervention | Patient  Community  Health system |
| Number of people seen in the intervention |  |
| Primordial prevention component  Yes  No  Unclear | If yes, provide details: |
| Primary prevention component  Yes  No  Unclear | If yes, provide details: |
| Secondary prevention component  Yes  No  Unclear | If yes, provide details: |
| Intervention inputs/resources used  When possible, provide quantitative data, e.g., budget of US$ 100,000 per year; 30 nurses trained during the intervention’s duration | Leadership/administration  Unclear/not stated |
|  | Financing – source(s) and amount(s)  Unclear/not stated |
|  | Healthcare workers (types and numbers)  Unclear/not stated |
|  | Technologies (drugs, diagnostic, etc)  Unclear/not stated |
|  | Information systems (medical record, etc)  Unclear/not stated |
|  | Systems of care delivery (guidelines, protocols, etc)  Unclear/not stated |

**II. Intervention results**

|  | Description as stated in the paper/report |
| --- | --- |
| Intervention health outcomes – quantify when possible | |
| Primordial prevention component |  |
| Primary prevention component |  |
| Secondary prevention component |  |
| Impact (change in health of targeted population) – experimental and quasi-experimental designs | |
| Primordial prevention component |  |
| Primary prevention component |  |
| Secondary prevention component |  |

**Section E: Practices performed in the intervention**

| Practices/activities | Description |
| --- | --- |
| **Primordial prevention** |  |
| Reduction in poverty, crowded living conditions, inequality  Yes  No  Unclear |  |
| Improving/expanding access to appropriate healthcare  Yes  No  Unclear |  |
| Others… |  |
| **Primary prevention** |  |
| Treating streptococcal A infections with antibiotics  Yes  No  Unclear |  |
| Community education and engagement  Yes  No  Unclear |  |
| Active case finding (sore throat clinics)  Yes  No  Unclear |  |
| Others… |  |
| **Secondary prevention** |  |
| Improve healthcare workers’ awareness  Yes  No  Unclear |  |
| Improve diagnosis and referral of RF and RHD  Yes  No  Unclear |  |
| Establishing local or central referral centre  Yes  No  Unclear |  |
| Registers of people living with RF/RHD  Yes  No  Unclear |  |
| Regular antibiotics for people at risk of RF recurrence  Yes  No  Unclear |  |
| Priority based follow up for people living with RF/RHD  Yes  No  Unclear |  |
| Others… |  |

**Objective two:**

**Section F: Emerging practices**

| **Notes:** |
| --- |
